# A Novel Familial Mutation in the *PCSK1* Gene That Alters the Oxyanion Hole Residue of Proprotein Convertase 1/3 and Impairs Its Enzymatic Activity

**DOI:** 10.1371/journal.pone.0108878

**Published:** 2014-10-01

**Authors:** Michael Wilschanski, Montaser Abbasi, Elias Blanco, Iris Lindberg, Michael Yourshaw, David Zangen, Itai Berger, Eyal Shteyer, Orit Pappo, Benjamin Bar-Oz, Martin G. Martín, Orly Elpeleg

**Affiliations:** 1 Gastroenterology Unit, Division of Pediatrics, Hadassah Hebrew University Hospital, Jerusalem, Israel; 2 Department of Anatomy and Neurobiology, University of Maryland-Baltimore, Baltimore, Maryland, United States of America; 3 Department of Pediatrics, Division of Gastroenterology and Nutrition, Mattel Children's Hospital and the David Geffen School of Medicine, University of California Los Angeles, Los Angeles, California, United States of America; 4 Endocrinology Unit, Division of Pediatrics, Hadassah Hebrew University Hospital, Jerusalem, Israel; 5 Neurology Unit, Division of Pediatrics, Hadassah Hebrew University Hospital, Jerusalem, Israel; 6 Department of Pathology, Hadassah Hebrew University Hospital, Jerusalem, Israel; 7 Department of Neonatology, Hadassah Hebrew University Hospital, Jerusalem, Israel; 8 Monique and Jacques Roboh Department of Genetic Research, Hadassah Hebrew University Hospital, Jerusalem, Israel; University of Iowa, United States of America

## Abstract

Four siblings presented with congenital diarrhea and various endocrinopathies. Exome sequencing and homozygosity mapping identified five regions, comprising 337 protein-coding genes that were shared by three affected siblings. Exome sequencing identified a novel homozygous N309K mutation in the proprotein convertase subtilisin/kexin type 1 (*PCSK1*) gene, encoding the neuroendocrine convertase 1 precursor (PC1/3) which was recently reported as a cause of Congenital Diarrhea Disorder (CDD). The *PCSK1* mutation affected the oxyanion hole transition state-stabilizing amino acid within the active site, which is critical for appropriate proprotein maturation and enzyme activity. Unexpectedly, the N309K mutant protein exhibited normal, though slowed, prodomain removal and was secreted from both HEK293 and Neuro2A cells. However, the secreted enzyme showed no catalytic activity, and was not processed into the 66 kDa form. We conclude that the N309K enzyme is able to cleave its own propeptide but is catalytically inert against *in trans* substrates, and that this variant accounts for the enteric and systemic endocrinopathies seen in this large consanguineous kindred.

## Introduction

Congenital diarrheal disorders (CDDs) are a group of devastating and potentially fatal neonatal enteropathies that often require parenteral nutrition. Recently Canani *et al.* [1] proposed classifying these disorders into 4 groups: 1) defects in digestion, absorption, and transport of nutrients and electrolytes, such as glucose-galactose malabsorption or sucrase-isomaltase deficiency, caused by mutations in *SLC5A1* [2] and *SI* [3] respectively; 2) disorders of enterocyte differentiation and polarization, such as microvillus inclusion disease and tufting enteropathy, caused by mutations in *MYO5B* [4] and *EPCAM* [5] respectively; 3) dysregulation of the intestinal immune response, as in immune dysregulation, polyendocrinopathy, enteropathy, X-linked (IPEX) syndrome caused by mutations in *FOXP3* [6] and 4) defects of enteroendocrine cell differentiation, as may be caused, for example, by mutations in *NEUROG3* [7]. These patients frequently endure a complex and costly diagnostic odyssey that often fails to produce a definitive diagnosis. Once infection is ruled out, in most cases the disorder is found to be inherited in an autosomal recessive manner; mutations in any of a large number of genes may be responsible [8]. Identification of a causal mutation can lead to improved management of the disease but genome-wide screening for mutations has not yet entered standard practice [9].

Mild mutations in the *PCSK1* gene (proprotein convertase subtilisin/kexin type 1) are associated with obesity [10], and more severe, rare mutations have increasingly been recognized as a cause of malabsorptive diarrhea and other endocrinopathies in a disorder called proprotein convertase 1/3 (PC1/3) deficiency (OMIM: 600955) [11]. The enzyme encoded by *PCSK1*, the neuroendocrine convertase 1 precursor, cleaves itself into the active form, neuroendocrine convertase 1 (also called prohormone convertase 1, proprotein convertase 1, and PC1), which is responsible for processing multiple peptide hormones within the enteroendocrine cell. Other rare heterozygous mutations in *PCSK1* have been observed in the general population, which likely would be harmful in an individual with two damaged copies of the gene [12]. PC1/3 deficiency involves a significant risk of mortality and failure to thrive secondary to severe generalized malabsorptive diarrhea in early childhood. However, the requirement for parenteral nutritional support decreases after 18 months of age, while various major systemic endocrinopathies develop [9], [11], [13]–[15]. To date, 17 individuals in 15 families have been reported to have disease-causing mutations in *PCSK1*. Thus enteroendocrine cell dysfunction governs the early clinical phenotype, while malabsorption may actually lessen the severity of the obesity that develops at later ages. Growth hormone deficiency, adrenal insufficiency, central diabetes insipidus, and hypogonadism are commonly observed [11]. Here, we describe four siblings with PC1/3 deficiency resulting from a novel mutation in the *PCSK1* gene.

## Material and Methods

### Subjects

The study was approved by the ethical IRB of Hadassah Hebrew University Medical Center and the parents signed informed consent.

### Genomic DNA Isolation

Genomic DNA was extracted from blood by standard procedures.

### Linkage analysis

A search for common homozygous regions in the DNA samples of affected patients was performed, using Affymetrix GeneChip Human Mapping 250K Nsp Array, as previously described [16].

### Whole exome analysis

**Figure 1:**
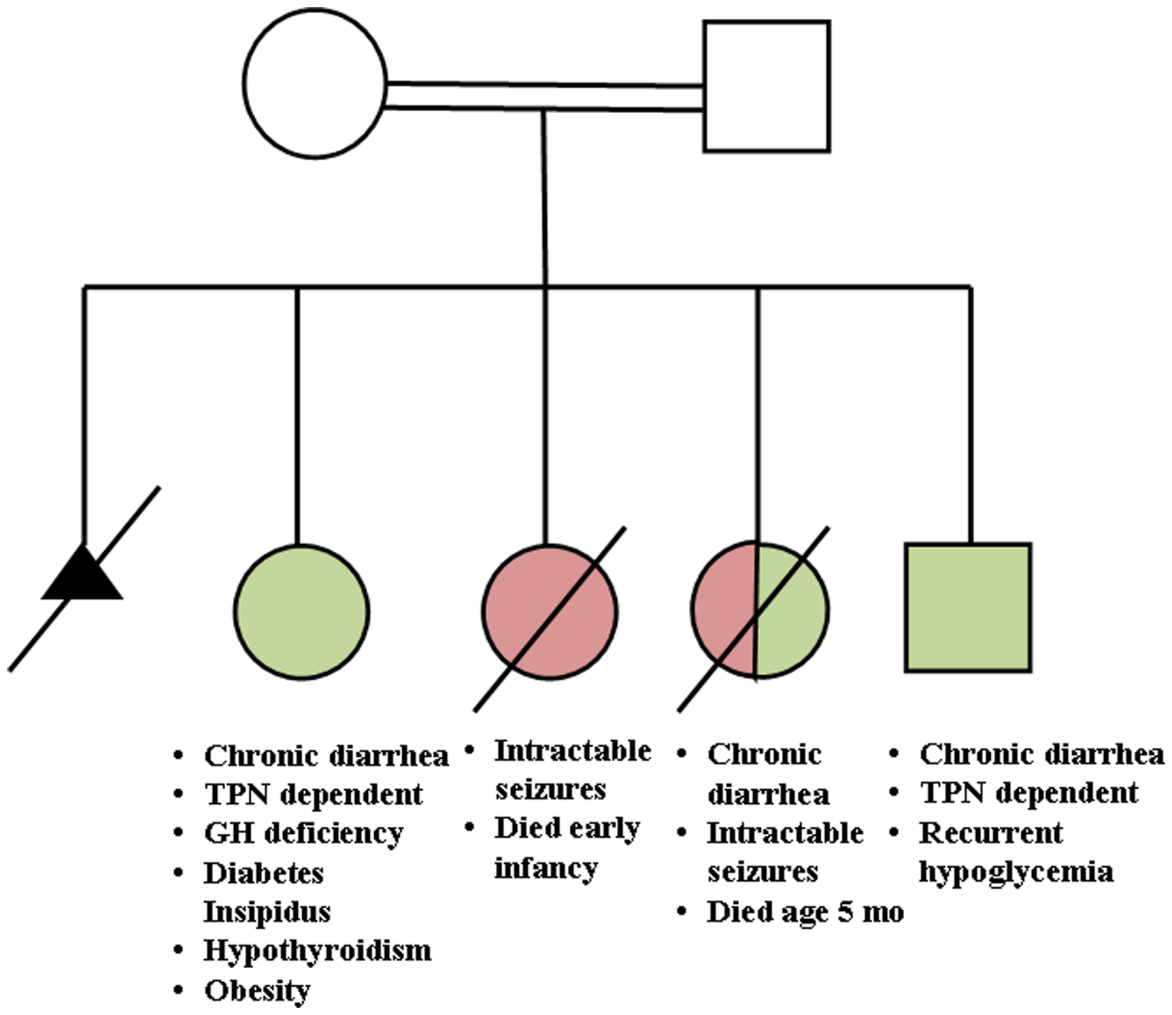
Family Pedigree. Pedigree showing intractable diarrhea (green) and seizures (red) and other clinical phenotypes. A slash through the symbol indicates that the subject is deceased, and a double line between the parents indicates a consanguineous union.

Protein coding exon sequences were enriched in the DNA sample of patient II-5 (fig. 1) using the SureSelect Human All Exon 50 Mb Kit (Agilent Technologies, Santa Clara, CA, USA). 100 base, paired-end sequences were read by HiSeq2000 (Illumina, San Diego, CA, USA). Reads alignment and variant calling were performed with DNAnexus software (Palo Alto, CA) using the default parameters with the human genome assembly hg19 (GRCh37) as a reference.

### Transient transfection of expression vectors

A wild-type human PC1/3 (NP_000430.3)-encoding plasmid, with a Flag-tag sequence inserted between the prodomain and the catalytic domain, was mutated at residue Asn309 to encode Lys (N309K AAC = >AAG; Genscript, Piscataway NJ) and verified by sequencing in its entirety. No other mutations were present. The wild-type and mutant plasmids were transiently transfected into either HEK293 or Neuro2A cells using FuGene (Promega, Madison WI); two days later, the overnight conditioned Optimem (containing 100 µg/ml aprotinin) was assessed for protein expression by Western blotting and was tested for enzymatic activity using a fluorogenic substrate, pERTKR-AMC (Peptides International, Lexington, KY), as previously described [11]–[12].

#### Pulse-chase experiments

Neuro2A cells were transfected with either wild-type or mutant PC1/3 vectors and labeled with ^35^S-methionine for 20 min, as described in Blanco et al. [17]. Cells were then chased for 2 h in medium containing cold methionine and lysed in boiling buffer for immunoprecipitation using N-terminal PC1/3 antisera (2B5) plus C-terminal PC1/3 antisera (3BF), and chase media were also immunoprecipitated. Immunoprecipitates were separated on 15% SDS-polyacrylamide gels, dried, and subjected to phosphoimaging.

### Histology

Small bowel mucosal biopsies from subjects II-2 and II-5 were stained by standard hematoxylin and eosin.

## Results

### Clinical Phenotype

Following one early first trimester miscarriage, four children were born at normal birth weight to healthy parents, who were first cousins of Moslem Arab ethnicity. The first child (II-2) developed severe malabsorptive diarrhea during first week of life with steatorrhea but without evidence of a protein-losing enteropathy (Fig. 1). Various dietary manipulations were attempted including hydrosylate and amino acid based formulae which failed. She was started after the first month of life on parenteral nutrition (PN), which was continued until five years of age.

**Figure 2:**
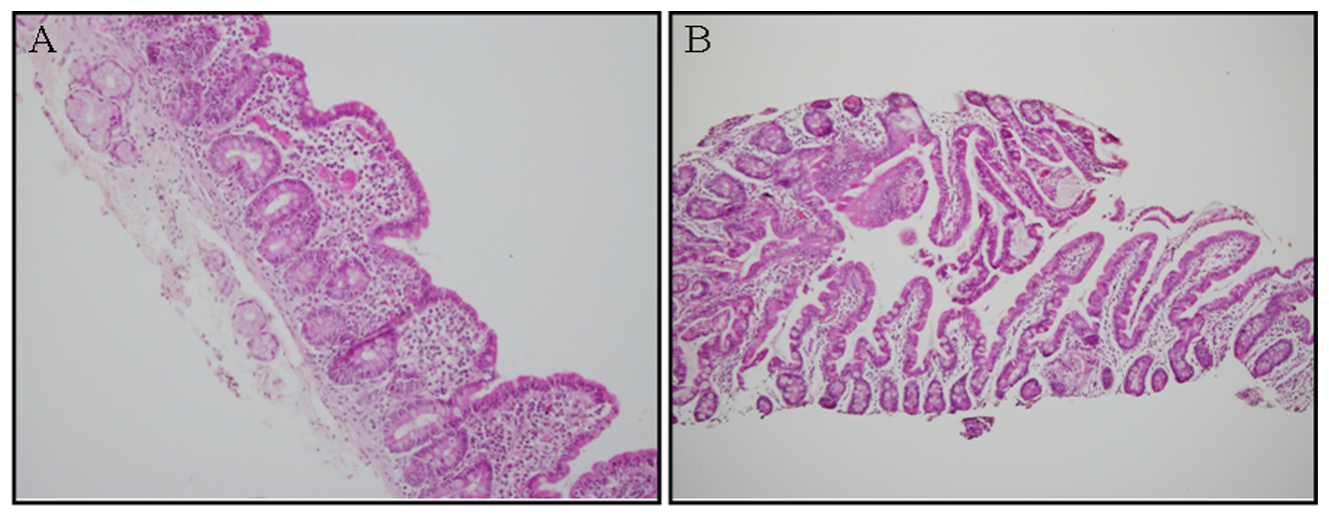
H&E staining of small bowel biopsies from patients II-2 and II-5. A) Mild villous atrophy associated with a slight increase in number of intraepithelial lymphocytes, and mild chronic inflammation of lamina propria (electron microscopy not shown-normal microvillous architecture); B) the villi appear normal without lymphocytic abnormalities in the intraepithelial and lamina propria compartments. Moreover, very few plasma cells were seen, and the epithelium was devoid of abnormal apoptosis, or abnormalities of microvilli.

A residual metabolic acidosis, resulting most probably from the chronic diarrhea, was treated by bicarbonate until eight years of age. Repeated small intestinal biopsies showed non-specific enteropathy including mild villous atrophy, slight increase in number of intraepithelial lymphocytes, and chronic inflammation of lamina propria (Fig. 2). The usual serologies indicative of celiac disease were undetectable.

Initially her relatively low FT4 levels were attributed to sick euthyroid syndrome, but as they persisted into her second year of life in spite of an improving clinical condition she was diagnosed with mild central hypothyroidism (TSH levels of 2–3 mIU/ml despite low free T4 levels of 7–9 pmol/l) and received thyroid replacement therapy. Given her poor growth rate and decreased peak GH response in 2 stimulation tests (only 3.5 and 6.5 ng/ml) by 3–4 years of age she did also receive GH replacement therapy that resulted in adequate growth rate with a height of 156 cm at 12 years of age and breast and pubic hair development at Tanner stage 3–4.

Other remitting endocrinopathies included self-limited episodes of diabetes insipidus (DI) with polyuria, a low urine osmolarity (70–150 mOsm/l), and elevated serum osmolarity (>300 mOsm/l). These episodes ended spontaneously with a short course of intranasal desmopressin (ddAVP) treatment. Finally, a gradual weight gain was initially attributed to a better appetite. However, she became increasingly severely obese by six years of age (Body Mass Index (BMI) –21.6), (50^th^ percentile 15.4) and continued to become more obese despite adequate GH and thyroid replacement, and intensive nutritional therapeutic guidance. Her neurologic development is normal and she has no convulsive disorder.

The two subsequent sisters (II-3, 4) were born after uneventful pregnancies and deliveries, but died at 9 days and 5 months of age respectively suffering from intractable seizures with left hemisphere rhythmic epileptiform activity by EEG. MR/MRS (at the age of 5 months) revealed periventricular white matter volume loss with ventricular dilatation. The second of these children had also recurrent episodes of diarrhea and hypothyroidism, and were treated with PN and thyroxine. Brain ultrasound scan in this infant was normal. The seizures were of multiple type (mainly clonic and myoclonic types) and unresponsive to all anti-epileptic drugs in various combinations. Both epileptic patients have neither permanent electrolyte nor metabolic imbalances associated with diarrhea. Epilepsy is an unexpected finding in patients with CDD especially in the absence of significant electrolyte or metabolic imbalances.

The youngest sibling (II-5) is now a one-year-old male who was born at normal birth weight following an uneventful pregnancy. He presented with neonatal diarrhea reminiscent of his two older sisters and required home PN for one year. No endocrinopathies or convulsive disorders have been found thus far and his small bowel biopsy showed only non-specific changes.

### Sequencing/Bioinformatics

The normal intestinal absorption of patient II-3, who suffered from severe epilepsy, suggested the presence of two non-linked disorders in the family, intractable diarrhea and drug-resistant epilepsy, both of which may have been transmitted in an autosomal-recessive manner, given the parental consanguinity. In order to identify the diarrhea-causing gene, we genotyped 250K SNPs in patients II-2 and II-4 who had intractable diarrhea and in patient II-3 who had normal bowel movement. This analysis resulted in the identification of five homozygous regions (Table 1) where the SNP genotype of patients II-2 and II-4 was identical and differed from that of patient II-3.

**Table 1:** Homozygous regions linked to the enteric disease in the family.

chromosome	start	end	size (Mb)
2	18294643	30607011	12.31
3	178985197	190692992	11.71
5	89679361	109389770	19.71
10	87890424	95523820	7.63
13	19625269	24664802	5.04

These regions encompassed 337 protein-coding genes and we therefore opted for whole exome sequencing of the DNA of patient II-5. The average coverage of the exons within the linked regions was 63X and 97.1% of the exons were covered >7X. Altogether we found 1089 missense or indel variants (only 46 of which were homozygous) that were not present in dbSNP version 129 or in the in-house dbSNP. Only four missense variants were located within the shared homozygosity blocks (Table 2).

**Table 2:** Homozygous variants within the diarrhea-linked regions of patient II-5.

Gene	HADHA	GTF3C2	PCSK1	FBXL17
**CHROM**	2	2	5	5
**POS**	26453084	27566454	95746646	107521954
**ID**	rs71441018	rs112001928	-	-
**REF**	C	A	G	G
**ALT**	G	G	C	A
**Transcript**	ENST00000380649	ENST00000359541	ENST00000311106	ENST00000542267
**cDNA**	c.652G>C	c.-24-9T>C	c.927C>G	c.1615-6C>T
**Protein**	p.Val218Leu	-	p.Asn309Lys	-
**Exon**	7/20	intron 1/18	8/14	intron 5/8
**Amino acid**	218/763	-/911	309/753	-/701
**Consequence**	missense_variant	intron_variant	missense_variant	splice_region_variant, intron_variant
**Gene name**	hydroxyacyl-CoA dehydrogenase/3-ketoacyl-CoA thiolase/enoyl-CoA hydratase (trifunctional protein), alpha subunit	general transcription factor IIIC, polypeptide 2, beta 110kDa	proprotein convertase subtilisin/kexin type 1	F-box and leucine-rich repeat protein 17
**OMIM**	LCHAD deficiency (609016; Trifunctional protein deficiency (609015)	-	Obesity and endocrinopathy due to impaired processing of prohormones (600955)	-
**CAROL^24^**	deleterious(1)	-	deleterious(1)	-
**Condel^23^**	deleterious(0.842)	-	deleterious(1)	-
**LRT^20^**	deleterious(1.03E-08)	-	deleterious(1.25E-10)	-
**Mutation Assessor^21^**	medium(2.76)	-	high(4)	-
**Mutation Taster^22^**	disease causing(1)	disease causing(0.98)	disease causing(1)	disease causing(0.85)
**PolyPhen2^19^**	probably_damaging(0.999)	-	probably_damaging(1)	-
**SIFT^18^**	deleterious(0.01)	-	deleterious(0)	-
**African American AF(hom)** *	0(0/2203)	0.003(0/2203)	0(2203)	-
**European American AF(hom)** *	0.000465(0/4300)	0.023(3/4297)	0(4300)	-

* Allele frequency (number of homozygous individuals) in the Exome Variant Server, NHLBI Exome Sequencing Project (ESP)^26^.

REF – GRCh37 reference allele, ALT – variant allele, OMIM – Online Mendelian Inheritance in Man^36^.

One of these variants, the N309K mutation in the *PCSK1* gene, appeared to be the probable cause of the CDD and endocrinopathies, given that other mutations in *PCSK1* are known to cause similar symptoms, whereas the other three variants (HADHA, FBXL17 and GTF3C2) are not known to be involved in CDD. The N309K mutation in the *PCSK1* gene was predicted to be deleterious by multiple *in silico* methods: SIFT [18] , PolyPhen2 [19], LRT [20], MutationAssessor [21], MutationTaster [22], Condel [23], and CAROL [24]. The mutation was not reported in the 1092 genomes of the 1000 Genomes project [25] nor in the 6503 exomes in the NHLBI Exome Sequencing project [26] (Table 2) Moreover the probability of splicing changes due to the intronic variants in FBXL17 and GTF3C2 was predicted to be low. Interestingly, the rare, but predicted as deleterious HADHA variant is associated with long-chain 3-hydroxyl-CoA dehydrogenase (LCHAD) deficiency (OMIM: 609016), but malabsorptive diarrhea is not associated with this condition.

**Figure 3:**
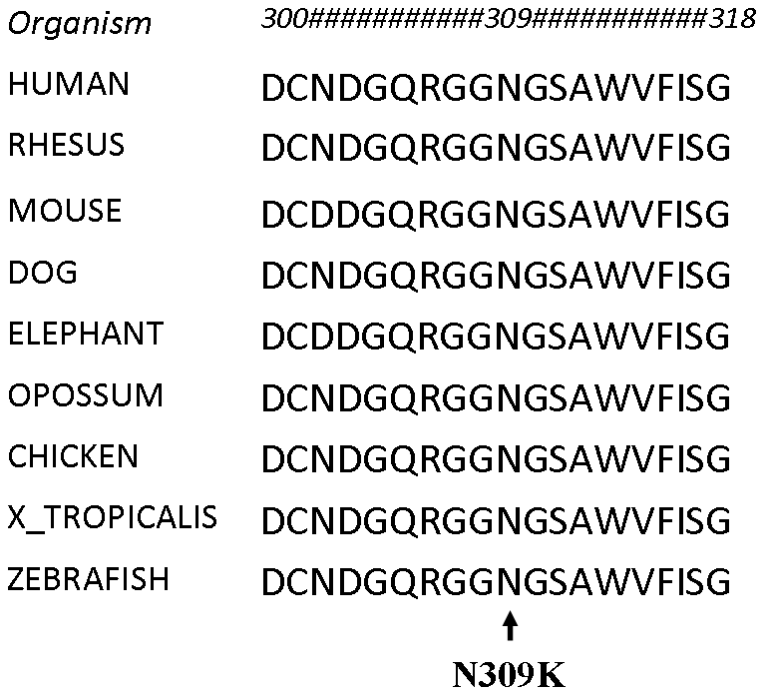
Alignment of N309K missense variant to members of the PC1/3 gene family. The location of the missense mutation within a conserved region in the catalytic domain of the PC1/3 gene family.

**Figure 4:**
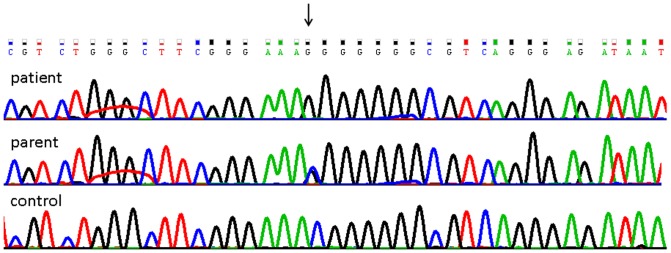
Sanger sequencing validation of the N309K variant. Sanger sequencing of the results for the proband, parent and unaffected control at nucleotide position 5: 95746646.

The enzyme encoded by *PCSK1*, prohormone convertase 1/3 (PC1/3), processes latent precursors to peptide hormones into their biologically active products within the enteroendocrine cell. The Asn residue at position 309 in the PC1/3 active site (the peptidase S8 family domain) is conserved in all vertebrates (Fig 3). Sanger sequencing of other affected and unaffected family members confirmed that this mutation segregated in the family, with patients II-2, II-4 and II-5 being homozygous and the parents (I-1 and I-2) and patient II-3 heterozygous (Fig 4).

### Functional Analysis and *In Vitro* Assessment

**Figure 5:**
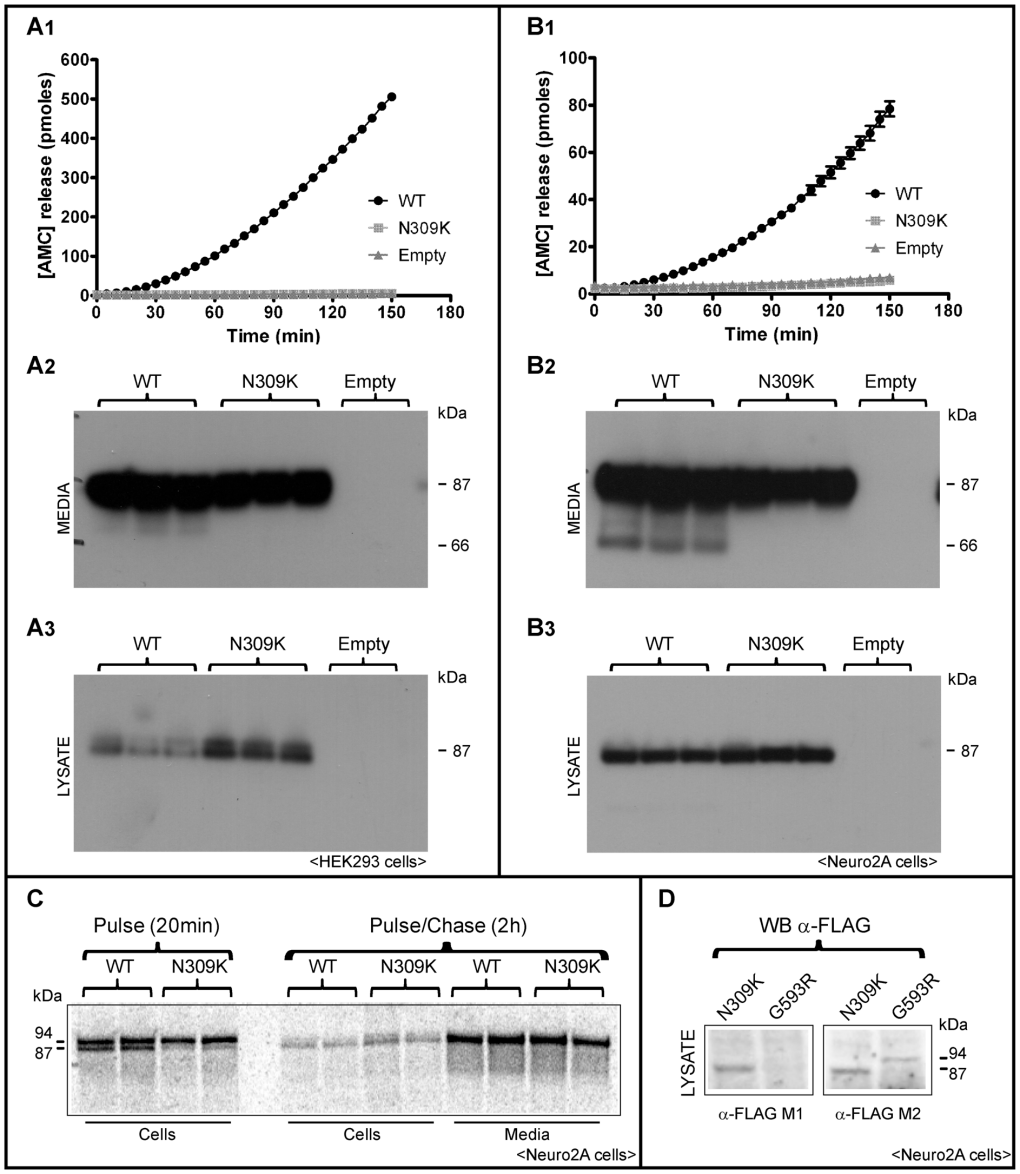
Lack of activity of N309K PC1/3 despite robust secretion. PC/3-encoding vectors were transfected into HEK (A panels, *left side*) or Neuro2A cells (B panels, *right side*) and the conditioned media subjected to either enzymatic assay (A1, B1) using the standard fluorogenic assay; or to Western blotting using PC1/3 antiserum (A2, B2). Cell extracts were also subjected to Western blotting (A3, B3). Panel C shows the maturation of ^35^S-methionine-labeled wild-type and N309K precursor proteins during a 20-minute pulse followed by a 2 h chase in Neuro2A cells. WT, wild-type PC1/3. Panel D shows a Western blot of Neuro2A cell lysate, previously transfected with either N309K and G593R PC1/3 cDNAs and incubated with α-FLAG M1 or α-FLAG M2 antibodies to discriminate the 94 kDa PC1/3 prodomain ER-retained form.

The position of this mutation corresponds to a residue critical to proprotein maturation and enzyme activity, the oxyanion hole transition state-stabilizing amino acid Asn309. To validate the functional significance of this rare variant, we expressed the wild type and the mutant PC1/3 protein in HEK293 and Neuro2A cells and tested its activity. Western blotting indicated that the N309K mutant protein exhibited apparently normal prodomain removal and was efficiently secreted from both HEK293 and Neuro2A cells (Fig 5). However, the secreted enzyme showed no catalytic activity (panels A1–B1) and was not processed into the 66 kDa form independently of cell context (panels A2–B3), indicating no *in trans* activity.

In order to better assess prodomain removal, we performed radiolabeling experiments of *PCSK1*-transfected Neuro 2A cells. Fig 5, Panel C shows that the N309K mutant underwent only slight prodomain removal (prodomain  = 94 kDa form; mature form  = 87 kDa) within the 20 minute pulse period, but showed complete prodomain removal within the 2 h chase period in media samples. In agreement with the Western blots, the mature 87 kDa mature form of the N309K mutant was secreted. These results contrast with results obtained using the G593R *PCSK1* mutant, which is totally unable to remove its own propeptide and is retained in the endoplasmic reticulum [15]. In lysate samples from Neuro2A transfected cells, the G593R proprotein was unreactive against the M1 Flag antiserum (which is specific for N-terminal Flag sequences) but reacted with the M2 Flag antiserum which recognizes internal Flag sequences, while the N309K protein reacted with both antisera (panel D). These data corroborate the removal of the propeptide in N309K-transfected cells.

## Discussion

The natural history of these children is consistent with the emerging clinical phenotype of proprotein convertase 1/3 deficiency, which typically involves CDD and an array of systemic endocrinopathies that develop in an age-dependent manner [11]. Neonates have severe generalized malabsorptive diarrhea and failure to thrive, and require prolonged total parenteral nutrition. As the disease progresses additional endocrine abnormalities develop, including diabetes insipidus, growth hormone deficiency, primary hypogonadism, adrenal insufficiency, and hypothyroidism. Moderate obesity, associated with severe polyphagia, generally appears despite early growth abnormalities. Patient II-2 required parenteral nutrition, and was diagnosed with diabetes insipidus, hypothyroidism and developed obesity in concordance with this disease phenotype [11].

In approximately 10–15% of infants who suffer from severe neonatal seizures the etiology is unknown, and it is assumed that these patients have genetic encephalopathies [27]. Several studies have elucidated the pathogenic role of genetic mutations involved in synaptogenesis, pruning, neuronal migration and differentiation, neurotransmitter synthesis and release, and structure and function of membrane receptors and transporters, but *PCSK1* variants have not been associated with a significant neurologic impairment [28]–[29].

*PCSK1* point mutations associated with PC1/3 deficiency result in the absence or significant reduction of secreted PC1/3 enzymatic activity similar to that observed here [9], [11]–[12]. However, the mutation presented herein, unlike previous reported cases, resulted in a secreted enzyme in which the propeptide was removed but no activity was present against synthetic substrates. The catalytic triad in eukaryotic subtilases consists of His-Ser-Asp; in addition, an Asn residue, which occupies a catalytic pocket site known as the oxyanion hole, is thought to stabilize the transition state of the product in both bacterial and eukaryotic subtilases [30]. Nearly all eukaryotic subtilases contain an Asn in this position; however, the prohormone convertase PC2 contains an Asp at this site. Substitution of the oxyanion hole Asn for Asp in the yeast subtilase kex2 resulted in a marked decrease in enzymatic activity [31]. However, substitution of the unusual Asp in PC2 with Asn did not result in measurable changes in enzyme activity [32], suggesting some flexibility in the oxyanion hole residue within prohormone convertases. By contrast, substitution of other catalytic residues in either PC1/3 or PC2 results in lack of prodomain removal, lack of secretion, and consequent loss of enzymatic activity [32]–[34].

The fact that the N309K mutant undergoes slowed prodomain removal implies that the identity of the oxyanion hole residue contributes to this intramolecular cleavage event. Interestingly, normal prodomain removal of a mouse PC1/3 N309D mutant was observed in *in vitro* translational experiments; in this work, potential effects of this substitution on enzymatic activity using other substrates were not studied [35].

We conclude that replacing the oxyanion hole residue (N309) by Asp [35]. or Lys (this study) does not suppress prodomain removal, and PC1/3 continues to traffic through the secretory pathway. Nevertheless, the complete loss of activity of the resultant mature mutant enzyme supports a critical role for the oxyanion hole residue in intermolecular substrate catalysis. The total absence of the PC1/3 66 kDa form in the medium secreted from Neuro2A cells transfected with the N309K mutant corroborates the idea that this PC1/3 variant is unable to cleave its own C-terminal domain in the usual intermolecular interaction [36]. These unequivocal effects on enzyme activity would be expected to result in the loss of all PC1/3-mediated processing in patients with this oxyanion substitution.
